# Lumbar Range of Motion Using the Wolfson Modified Schober Test

**DOI:** 10.5435/JAAOSGlobal-D-24-00041

**Published:** 2024-07-10

**Authors:** Raphael Lotan, Lev Klatzkin, Itzik Lan, Mojahed Sakhnini, Oded Hershkovich

**Affiliations:** From the Department of Orthopedic Surgery, Wolfson Medical Center, Holon, Israel (Dr. Lotan, Dr. Klatzkin, Dr. Lan, and Dr. Hershkovich), affiliated to the School of Medicine, Tel Aviv, Israel (Dr. Lotan, Dr. Klatzkin, Dr. Lan, and Dr. Hershkovich), and the Department of Orthopedic Surgery, Ziv Medical Center, Zfat, Israel (Dr. Sakhnini), affiliated to the Bar Ilan University, Bnei Brak, Israel (Dr. Sakhnini).

## Abstract

**Introduction:**

Lumbar range of motion (ROM) is a critical component of spinal function and often affected by age and sex. This study aimed to evaluate the variations in lumbar ROM across different age groups in a healthy adult population and determine the influence of sex, height, weight, and body mass index.

**Methods:**

A total of 208 subjects (106 men, 102 women) were recruited and stratified into age groups from the 20s to 60s and older. Lumbar ROM was measured using the Wolfson modified Schober test. Data were analyzed for flexion, extension, and total ROM. Linear regression examined the predictors of lumbar ROM.

**Results:**

The study found a progressive decline in lumbar flexion and total ROM with age. Age was the only notable predictor of lumbar flexion, with no notable effect of weight and body mass index on ROM. Extension measurements were inconsistent and did not show a clear pattern across age groups.

**Discussion:**

Age-related changes in lumbar ROM were consistent with known physiological changes within the spine. Despite physical differences in height and weight, the lumbar spine ROM was similar between sexes, highlighting the influence of age over sex in lumbar motion. Lumbar ROM decreases with age, with flexion affected more than extension.

Evaluating the range of motion (ROM) is crucial in monitoring spine patients, assessing the effect of various therapies, and planning subsequent treatment strategies.^1,2,3^ Still, it should not be used alone when aiming to quantify disability.^1^ However, ROM estimation remains vital to detecting impairment and integral to treatment planning.^4,5,6^ Lumbar ROM is a crucial parameter in determining disability in the context of workers' compensation, insurance claims, and litigation worldwide.^7,8^ Lumbar ROM assessment methods should ensure accuracy, reliability, and repeatability.^9^

A few lumbar ROM measurement methodologies have been documented with appreciable reproducibility features in existing literature.^10^ Tools and methods currently used include the Draughtsman flexible ruler,^11,12,14^ finger-floor method,^15^ double inclinometer (DI) method,^16,17,19^ and tape measurement method,^19,20,25^ as well as specific devices like Cybex EDI-320 (electronic digital inclinometer) ^26,27,28^ and the Back ROM device (BROM II).^29^ The tape measurement and DI have demonstrated good reproducibility in measuring lumbar flexion ROM. However, the clinical application of DI is limited because of its demanding nature in terms of time and expertise.^19^ The tape measurement method is commonly used and requires no special equipment, skill, or training.

The original Schober^22^ test uses a tape over the spine between the lumbosacral junction and 10 cm above it. The challenge in precise localization of the lumbosacral junction led to an adaptation to the original test ^23^ by adding 5 cm below and 10 cm above the lumbosacral junction. When published, the modified Schober test (MST) was compared with L1-S1 radiographic measurements on a small group of patients and found a very high correlation. Additional studies reported high accuracy and reproducibility.^21,24,25^ Nonetheless, the use of the MST has been challenged in the past decade, with others reporting lower accuracy rates.^20,30^ In one study, 50 healthy subjects were evaluated for lumbar flexion, and the authors stated that only four spinal segments (L2-S1) were actually included in the MST measurement of lumbar spine flexion.^30^ Based on these findings, the utility of this method was questioned on both scientific and clinical grounds and echoed earlier criticism concerning the distance between skin landmarks.^20^ These authors used different distances between the posterior superior iliac spine (PSIS) and a midline landmark at 5, 10, 15, and 20 cm cephalad. They concluded that the 15-cm and 20-cm segments above the PSIS's midline contributed little to an overall measure of lumbar flexion. Based on these results, they proposed a second adaptation of the original Schober test, the modified–modified Schober test (MMST) using the 15-cm distance cranial to PSIS landmarks.

The MMST, the last modification of the Schober test, includes two improvements: (1) the use of the PSIS as opposed to the lumbosacral junction to include the L5-S1 movement while eliminating the difficulty of finding the lumbosacral junction and (2) a 15-cm cephalad landmark to include all lumbar motion segments. However, the reproducibility properties of the MMST needed a better anatomical establishment.

In a previous study,^9^ the Schober test and its variants, surface anatomical marker accuracy and repeatability, were compared with actual L1-S1 motion segmental length as measured on sagittal CT and the influence of patient sex and habitus on these measurements. The study proved that even with an accurate CT scan–based Schober test starting point, the 10-cm interval failed to include 68% of the L1-L2 motion segments and 32% of the L2-3 motion segment. Based on these results, the Schober test is highly inaccurate and should be abandoned entirely. The MST adds caudal length to measure nonmobile sacral segments without improving the measurements of the mobile lumbar segments. Therefore, this modification is inaccurate, as the Schober test should also be avoided. The MMST ^20^ bypassed the L5-S1 junction and relied on the PSIS bony landmark as an identifiable starting point with a 15-cm interval that was supposed to include the entire L1-S1 motion segments. The MMST markedly improved the L1-S1 segment measurement from 32% accuracy of the Schober/MST to 76%. The MMST still failed to include the L1-2 segment in 24% of patients, remaining a suboptimal assessment of the L1-S1 motion segments.

Using the MMST advantage of PSIS bony landmarks as a better anatomical anchor than the L5-S1 junction, Our team ^9^ reexamined the CT scans with a 16-cm interval length and found that this Wolfson modified Schober test (WMST) improved the accuracy of measuring L1-S1 segments to 96% with only one case, which also included the T12-L1 motion segment (Figure 1). Including the T12-L1 motion segment in the measurement has less effect on lumbar flexion-extension motion than disregarding the L1-2 motion segment, as seen in the MMST.^31^

**Figure 1:**
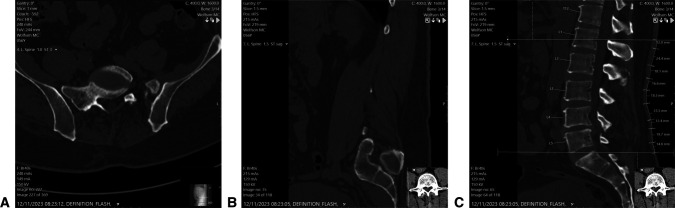
Radiographs demonstrating Wolfson modified Schober test measurements presented on CT. A**,** Radiograph demonstrating the PSIS landmark on CT axial and sagittal views. **B,** Radiograph demonstrating the PSIS caudal starting point. The PSIS tip was measured by marking the most prominent point of the PSIS, as measured by using axial and sagittal CT views, representing the PSIS as palpated on physical examination (marked arrow). On that level, scrolling back to a midline sagittal plane, a parallel line was drawn to the skin surface as described previously. **C,** Radiograph demonstrating WMST complete CT measurement. PSIS = posterior superior iliac spine, WMST = Wolfson modified Schober test.

This study aimed to evaluate the lumbar ROM in a healthy population according to sex, age, and body mass index (BMI), thus creating a reference tool for physicians and physiotherapists using the WMST.

## Methods

The study cohort included 200 healthy subjects aged 18 to 120 years, 100 men and 100 women, visiting the medical center for non–low back pain reasons. The cohort was preplanned to include 20 subjects of each sex in every decade of life. Exclusion criteria included age younger than 18, pregnancy, history of spine surgery, infection, tumor, fracture or malformation (scoliosis or kyphosis), and systemic inflammatory disease. We excluded subjects with a low back pain episode while including subjects with a previous episode of low back pain that did not require an imaging investigation or treatment other than medication. Professional athletes were excluded. Every subject was measured for height and weight. On every subject, while standing erect, a line connecting the PSIS and a line 16 cm cephalad to this line was drawn, as measured using a tape measurement. The subject was then asked to bend forward as much as possible, and a tape measured the distance between the lines. The same procedure was performed during maximal lumbar extension (using the described WMST for assessment). The investigators excluded subjects who reported pain during lumbar motion or were considered uncooperative.

All measurements were summed and coded for statistical analysis. R Statistical Software, version 3.5.2 (Foundation for Statistical Computing), was used to conduct statistical analyses. Correlations between each of the Schober test modifications and subjects’ sex, BMI, weight, height, and lumbar lordosis were examined using Student *t*-tests and linear regression.

The Institutional Review Board approved this study.

## Results

A total of 208 subjects, 106 men and 102 women, were recruited in this study. The cohort was grouped by age as 20s, 30s, 40s, 50s, 60s, and older while every subgroup had 19 to 23 subjects (Table 1).

**Table 1:** Cohort Data by Sex

	20s			30s			40s			50s			60+		
	Male	Female	*P*	Male	Female	*P*	Male	Female	*P*	Male	Female	*P*	Male	Female	*P*
n	23	20		21	20		22	19		20	21		20	22	
Age	26 ± 3.8	25.1 ± 3	0.342	35.7 ± 2.6	35 ± 3.2	0.465	43.9 ± 2.9	45.5 ± 3.5	0.134	54.8 ± 3.1	55.7 ± 2.4	0.302	70.3 ± 7.6	69 ± 5.8	0.568
Height	176.4 ± 7.1	163.4 ± 8.6	0.000	179.7 ± 7.5	164.8 ± 8.7	0.000	173 ± 6	163.6 ± 7.3	0.000	173.3 ± 6.2	165 ± 6.7	0.000	172.7 ± 8.5	160.6 ± 7.9	0.000
Weight	77.5 ± 19.1	66.8 ± 15.4	0.049	81 ± 13.6	64 ± 9.4	0.000	79.6 ± 15.8	66.1 ± 13.8	0.006	84.4 ± 15.4	73 ± 12.9	0.015	79.7 ± 14.5	73.7 ± 13.3	0.170
BMI	24.7 ± 4.9	24.9 ± 4.5	0.903	25.1 ± 4.1	23.5 ± 2.6	0.146	26.5 ± 4.7	24.8 ± 5.5	0.279	28 ± 4.4	26.9 ± 4.4	0.402	26.7 ± 4.5	28.8 ± 6.3	0.223
Flex	7.2 ± 1.3	7 ± 1.4	0.607	7.1 ± 1	6.7 ± 1.8	0.46	6.8 ± 0.9	6.4 ± 1.1	0.189	6 ± 1.8	6 ± 1	0.831	5.2 ± 1.7	4.5 ± 1.6	0.184
Ext	0.8 ± 0.4	0.8 ± 0.7	0.795	1.2 ± 0.7	1.2 ± 0.6	0.95	1.2 ± 0.8	0.9 ± 0.5	0.195	0.7 ± 0.5	0.7 ± 0.5	0.924	1.4 ± 0.7	1.3 ± 0.5	0.584
Total ROM	8.1 ± 1.4	7.8 ± 2	0.618	8.3 ± 1.4	8 ± 1.9	0.491	8 ± 1.4	7.3 ± 0.9	0.057	6.6 ± 1.9	6.7 ± 1	0.815	6.6 ± 1.5	5.8 ± 1.8	0.136

ROM = range of motion

The male cohort's age varied as expected across the groups, with the average age being 26 ± 3.8 years for the 20s, 35.7 ± 2.6 years for the 30s, 43.9 ± 2.9 years for the 40s, 54.8 ± 3.1 years for the 50s, and 70.3 ± 7.6 years for the 60+ age group. The average height showed minimal variation across the age groups. The tallest average height was observed in the 30s group at 179.7 ± 7.5 cm while the shortest was in the 50s group at 173.3 ± 6.2 cm. Weights varied from 77.5 ± 19.1 kg in the 20s group to 84.4 ± 15.4 kg in the 50s group. Body mass index values ranged from 24.7 ± 4.9 in the 20s to 28 ± 4.4 in the 50s. Lumbar flexion values, as well as total lumbar ROM, showed a progressive decline across age groups.

As with men, the female cohort's age varied consistently across groups. Heights ranged from 163.4 ± 8.6 cm in the 20s to 165 ± 6.7 cm in the 50s. Weights ranged from 64 ± 9.4 kg in the 30s to 73.7 ± 13.3 kg in the 60+ age group. Body mass index values showed a decrease from the 60+ group (28.8 ± 6.3) to the 20s group (24.9 ± 4.5). Lumbar flexion values, as well as total lumbar ROM, demonstrated a progressive decline across age groups.

When comparing male and female age distribution between the subgroups, the distribution was statistically similar (0.134 < *P* < 0.568) while height was statistically different between men and women in all age groups (*P* < 0.001). Weight was also significantly higher in men of all age groups (*P* < 0.05), except for the 60+ cohort (*P* = 0.17). Male and female BMI was similar in all age groups, respectively (0.146 < *P* < 0.903). Lumbar flexion, extension, and total ROM were not statistically different when comparing age groups (0.136 < *P* < 0.95). Given this similarity between men and women across age groups, Table 2 combines male and female data.

**Table 2:** Cohort Data

Age	20-29	30-39	40-49	50-59	60+
Average age	25.6 ± 3.4	35.3 ± 2.9	44.6 ± 3.3	55.2 ± 2.8	69.6 ± 6.6
Height (cm)	170.3 ± 10.2	172.4 ± 11	168.6 ± 8	169 ± 7.6	166.4 ± 10.1
Weight (kg)	72.5 ± 18.1	72.7 ± 14.5	73.3 ± 16.2	78.6 ± 15.1	76.5 ± 14
BMI	24.8 ± 4.7	24.3 ± 3.5	25.7 ± 5.1	27.4 ± 4.4	27.8 ± 5.5
Flexion (cm)	7.1 ± 1.3	6.9 ± 1.5	6.6 ± 1	6 ± 1.4	4.9 ± 1.6
Extension (cm)	0.8 ± 0.6	1.2 ± 0.7	1.1 ± 0.7	0.7 ± 0.5	1.3 ± 0.6
Total ROM	7.9 ± 1.7	8.1 ± 1.6	7.7 ± 1.2	6.7 ± 1.5	6.2 ± 1.7

ROM = range of motion

Table 2 summarizes that the flexion motion decreased from 7.1 ± 1.3 cm in the 20s to 4.9 ± 1.6 cm in the 60s and older, in a linear fashion. The same pattern was seen in the total lumbar ROM (from 7.9 ± 1.7 cm in the 20s to 6.2 ± 1.7 cm in the 60s and older age group). When examining men and women with similar height, the dependency on height remained unchanged.

Extension motion was markedly smaller than flexion in all age groups and sexes and showed erratic values in the different age groups.

**Figure 2:**
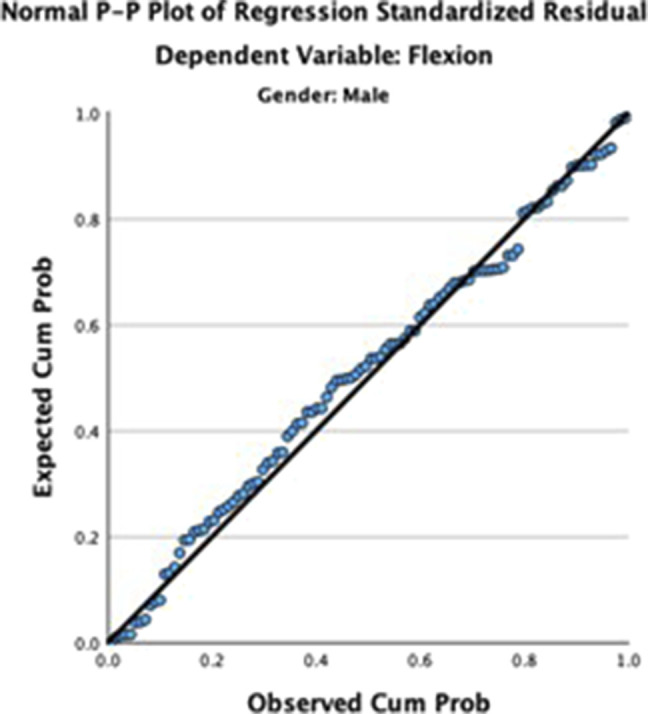
Plot demonstrating linear regression of lumbar flexion in men.

**Figure 3:**
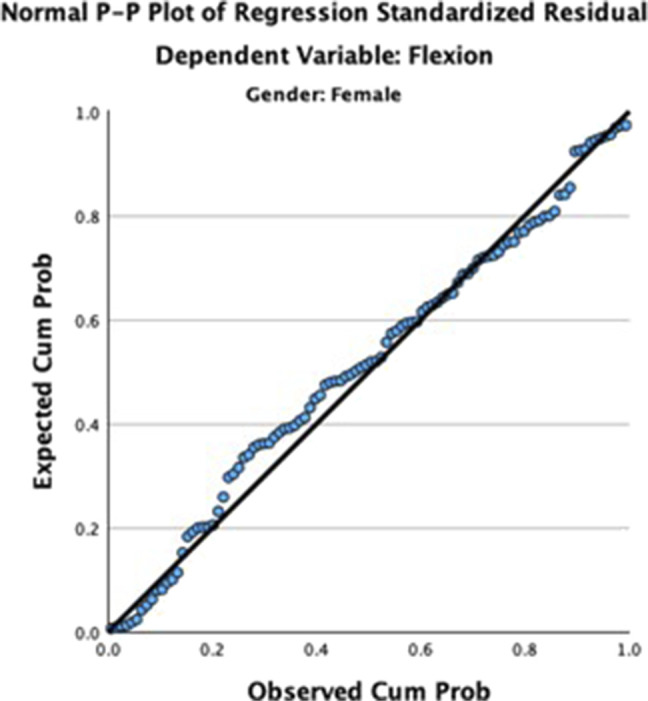
Plot demonstrating linear regression of lumbar flexion in women.

Using linear regression for male and female ROM separately (Figures 2 and 3), height and age were statistically significant for lumbar flexion in men (F = 15.5, R^2^ = 0.231, *P* < 0.001) while only age was significant for lumbar flexion in women (F = 32.2, R^2^ = 0.244, *P* < 0.001). When combining men and women, age was the only notable factor in predicting lumbar flexion, and the amount of flexion linearly reduced with age (Figures 2 and 3). Weight and BMI were not found to be relevant factors in lumbar flexion motion. Lumbar extension could not be predicted by age, height, weight, BMI, or sex.

According to the linear regression models, the predicted lumbar flexion for women is as follows:

## Flexion (cm) = 8.46*−*0.051 × (age)

The predicted lumbar flexion for men is as follows:

Flexion (cm) = 5.7 + 0.16 × (height)*−*0.043 × (age)

Alternatively, when excluding height, we have the following:

Flexion (cm) = 8.55*−*0.046 × (age)

## Discussion

In this study, the WMST was used to comprehensively analyze the lumbar ROM across different age groups. Our findings suggest distinct variations in the lumbar motion, particularly flexion, as subjects age. Moreover, while there were evident differences between men and women in some physical parameters like height and weight, the lumbar flexion and extension patterns remained consistent between sexes.

The age-wise distribution of the cohort was relatively balanced, which provides a robust basis for comparison. As anticipated, the male and female cohort's age varied consistently across groups. Interestingly, height displayed minimal fluctuation, 3 to 4 cm, across age groups for men and women, with the most notable deviation seen between the 30s and 50s groups, consistent with literature suggesting that adult height remains relatively constant after adolescence.^24,32,33^

With advancing age, one striking observation is the progressive decline in lumbar flexion values and total lumbar ROM. This phenomenon, noticed in both men and women, aligns with previous research highlighting age-related changes in the spine that could lead to decreased flexibility. Disk dehydration, facet joint osteoarthritis, and ligamentous thickening are known age-related changes that could impede spinal mobility.^24,32,33^

The comparison between male and female data reveals intriguing insights. While height and weight predictably differ between sexes (with men generally being taller and heavier), there was no notable difference in lumbar ROM. This might suggest that despite anatomical differences, the functional attributes of the spine related to motion may be conserved across sexes.

Furthermore, the linear regression analysis provided a deeper understanding of the factors affecting lumbar ROM. Age was a notable predictor for lumbar flexion in both sexes but more prominently in men when considered separately. The fact that age remains the sole notable predictor when combining data of both sexes further reinforces its role in spinal mobility. Conversely, weight and BMI, which might be intuitively linked to mobility because of the load on the spine, were not relevant in predicting lumbar flexion. This observation is intriguing and might suggest that intrinsic age-related spinal changes may have a more pronounced effect on flexibility than external weight-bearing factors.

The linear regression models allowed for a clear, simple, and validated tool for predicting lumbar flexion in both sexes. The predicted lumbar flexion for women is *Flexion (cm) = 8.46−0.051 × (age)*. The predicted lumbar flexion for men is *Flexion (cm) = 5.7 + 0.16 × (height)−0.043 × (age)*, or alternatively, when excluding height, *Flexion (cm) = 8.55−0.046 × (age)*. This simple equation provides a useful, reliable, reproducible tool for medico-legal purposes and clinical assessment.

The lumbar extension motion exhibited some unpredictability across age groups, and unlike flexion, its pattern could not be predicted by any of the parameters. This unpredictability might be due to individual variations or could be indicative of the biomechanical intricacies of extension compared with flexion.

This study had limitations. Sample distribution: While the study had a total of 208 subjects, they were not evenly distributed across age groups. Each age group had 19 to 23 subjects, which may not provide sufficient power for some statistical analyses, especially when comparing subgroups. Age bracketing: The cohort was grouped into specific age brackets as 20s, 30s, etc. This method might overlook nuances within each decade, as someone aged 20 and someone aged 29 might have notable differences despite being in the same bracket. Data for older age groups: The oldest age bracket is labeled as 60 and older, which potentially includes a wide range of ages (from 60 to 80 or older). Grouping these ages might obscure differences in the very elderly compared with those in their early 60s. Variable specificity: The study used broad metrics like height, weight, and BMI. No differentiation or consideration of factors like muscle mass, bone density, or fat percentage was observed, which could influence the results, especially when discussing lumbar flexion and ROM. Inconsistency in extension motion data: Extension motion displayed erratic values across different age groups. This variability might suggest potential measurement inconsistencies or could be a result of other unexplored factors. Scope of the study: The study focused solely on lumbar flexion and extension without considering other potential lumbar movements or factors that could influence these motions, such as rotation or lateral flexion. Lack of longitudinal data: The study appears to be cross-sectional, capturing data at a single point in time. Longitudinal data, observing changes in the same subjects over time, might provide a more comprehensive understanding of lumbar mobility changes across the lifespan. As with all Schober modifications, WMST does not measure rotation or lateral bending. Incorporating these limitations in future research could clarify lumbar mobility across different demographics and age groups.

In conclusion, our study underscores the importance of age as a determinant of lumbar flexibility, particularly in flexion. Using the previously CT-ascertained WMST, it provides the missing data regarding the normal lumbar ROM in a healthy population across decades. This study also suggests a novel, simple equation predicting lumbar flexion. The findings also emphasize that while there are distinct sex differences in height and weight, these do not markedly influence the functional aspects of lumbar ROM. Future studies could delve deeper into understanding the underlying biomechanical and histological changes in the aging spine that lead to these observations. The WMST should be accepted and used as the most anatomically accurate way of measuring lumbar flexion motion.
